# Major cardiovascular events in long-term multiple myeloma survivors: a Korean case–control study (the CAREMM-2105 study)

**DOI:** 10.1007/s10238-024-01368-2

**Published:** 2024-06-12

**Authors:** Jeonghoon Ha, Suein Choi, Seulji Moon, Jinseon Han, Jeongyoon Lee, Ki-Hyun Baek, Seunghoon Han, Sung-Soo Park, Chang-Ki Min

**Affiliations:** 1 Division of Endocrinology and Metabolism, Department of Internal Medicine, Seoul St. Mary’s Hospital, College of Medicine, The Catholic University of Korea, Seoul, 06591 Republic of Korea; 2 Department of Pharmacology, College of Medicine, The Catholic University of Korea, Seoul, 06591 Republic of Korea; 3 Pharmacometrics Institute for Practical Education and Training (PIPET), College of Medicine, The Catholic University of Korea, Seoul, 06591 Republic of Korea; 4 Division of Endocrinology and Metabolism, Department of Internal Medicine, Yeouido St. Mary’s Hospital, College of Medicine, The Catholic University of Korea, Seoul, 06591 Republic of Korea; 5 Seoul St. Mary’s Hematology Hospital, College of Medicine, The Catholic University of Korea, Seoul, 06591 Republic of Korea; 6 Leukemia Research Institute, The Catholic University of Korea, Seoul, 06591 Republic of Korea

**Keywords:** Multiple myeloma, Cardiovascular disease, Mortality, Survivors

## Abstract

**Purpose:**

Despite improvements in multiple myeloma (MM) survival rates, data on cardiovascular outcomes in long-term survivors remain lacking.

**Methods:**

This retrospective case–control study utilized the Korean National Health Insurance Service database (2009–2020) to compare the incidence of cardiovascular disease (CVD) between patients with MM and a matched control group, focusing on long-term (> 5 years) survivors. A preliminary case cohort (n = 15,402 patients with MM) and a matched control cohort (n = 123,216 patients without MM) were established based on birth year and sex. Following 1:1 propensity score matching, the final matched cohorts each comprised 15,402 participants.

**Results:**

The case and control cohorts were comparable in mean age (66.2 ± 11.5 years vs. 66.1 ± 11.3 years), sex, age distribution, and comorbidities. By the 8-year follow-up, the cumulative incidence of CV events (12.5% vs. 22.1%) and CVD risk were significantly lower in the case cohort. The 5-year landmark analysis revealed significant differences in CVD incidence between the cohorts (7.8% [case cohort] vs. 9.8% [control cohort]), with variations across age groups and sex, highlighting a significantly higher CVD risk among patients aged < 50 years in the case cohort (*P* < 0.001).

**Conclusions:**

These findings underscore the need for vigilant CVD monitoring in MM long-term survivors, particularly those aged < 50 years at first diagnosis.

**Implication for Cancer Survivors:**

This study highlights the importance of integrating cardiovascular monitoring and risk management into long-term care for MM survivors, with a focus on younger patients and personalized interventions.

## Introduction

Multiple myeloma (MM), the second most common hematologic malignancy worldwide, is characterized by the abnormal growth of plasma cells in the bone marrow [1–3]. In South Korea, the age-standardized incidence rate is 1.7 per 100,000 people, underscoring its significance compared with global averages [4]. The prevalence of MM increases with age; approximately 35% of patients are aged over 75 years, and an additional 10% are older than 85 years [5, 6]. Many of these older patients present with cardiovascular (CV) or cerebrovascular disease risk factors or comorbidities at the time of MM diagnosis [6, 7]. This renders the management of CV or cerebrovascular complications a key challenge, particularly in this age group [8]. Cardiovascular disease (CVD) ranks among the most common comorbidities in patients with MM and is a leading cause of mortality in Western countries [7].

Treatments for MM, often administered concurrently and each capable of elevating the risk of CV side effects, have increasingly been linked to cardiac comorbidities, despite their success in extending patient lifespans [9, 10]. Therefore, the assessment and management of CVD have emerged as crucial aspects in the care of patients with MM, especially in light of adverse effects, such as cardiotoxicity and arrhythmias, associated with medications used in the treatment of MM [11–13]. However, whether the prevalence of CVD continues to be higher among long-term survivors of MM remains uncertain. This is because patients with MM are often vigilant about the potential occurrence of such diseases and proactively engage in assessing and preventing CVD events. Whether long-term survivors who have undergone successful treatment still face an increased risk of CVD compared with those treated in the acute phase of MM remains particularly unclear.

Reports indicate CVD events in up to 7.5% of patients with MM, with a median overall survival of 47.6 months observed in phase 3 clinical trials [14]. However, real-world evidence regarding the patterns of these events among large patient populations and long-term MM survivors is lacking. In Korea, the 5-year survival rate has seen a significant increase from 23.5% in the period 1993–1995 to 46.6% in 2015–2018 [4]. As the number of long-term survivors grows, the need for information on CVD events occurring in long-term MM-survivors is increasing. Therefore, this study aimed to ascertain the survival rates of patients with MM and the incidence of CVD among long-term MM survivors, utilizing the latest real-world data from Korea.

## Methods

### Data source and study population

This study utilized data from the Korea National Health Insurance Service (KNHIS) database, which is a comprehensive healthcare information system that provides healthcare coverage to all South Korean individuals. The database contains health-related information for approximately 50 million individuals and includes sociodemographic data; lifestyle questionnaires; anthropometric measurements; laboratory test results; medical diagnoses based on the International Statistical Classification of Diseases, Tenth Revision, Clinical Modification (ICD-10-CM); and treatment data for the Korean population [15]. This study was approved by the Seoul St. Mary’s Hospital Institutional Review Board, Korea (approval number: KC21ZNSI0448) and adhered to the principles outlined in the Declaration of Helsinki, along with other applicable regulations and guidelines. Since the research involved the analysis of publicly accessible data that had been anonymized and de-identified, the requirement for obtaining informed consent was waived.

### Construction of study cohort

This retrospective case–control cohort study utilized the Korean National Health Insurance Service (KNHIS) database. We systematically identified individuals between January 1, 2009, and December 31, 2020. The case group consisted of patients diagnosed with MM based on the specific ICD-10 code (C90) as either a primary or secondary diagnosis. For the non-MM control group, which lacked a prior history of MM, we employed 1:10 age/sex-matching from the KNHIS database’s sample cohort. This sample cohort selects approximately 2% of samples directly from the entire Korean population database. This approach minimizes non-sampling errors.

Initially, we established a primitive case cohort (n = 15,402), excluding patients with MM for reasons, such as missing values (n = 332), age under 19 years (n = 52), MM as a secondary diagnosis (n = 13,432), exclusive diagnosis in 2009 or only once (n = 6396), or prior diagnosis of CVD before MM diagnosis (n = 2220). Similarly, we constructed a primitive control cohort (n = 123,216) without MM by 1:8 matching with the case cohort based on birth year and sex. This cohort was further refined by excluding individuals with missing values (n = 2405), those who died prior to the index date (n = 119), and those diagnosed with CVD before the index date (n = 13,442), resulting in a mother control cohort (n = 107,610). To balance the baseline characteristics of both cohorts, we applied 1:1 propensity score matching (PSM), adjusting for factors such as birth year, sex, index year, socioeconomic status, and comorbidities. Consequently, the final study cohort paired one MM patient with each non-MM control, resulting in 15,402 participants in both the MM (case cohort) and non-MM (control cohort) groups.

### Covariates

Data on baseline characteristics were collected at the index date. We utilized the Charlson Comorbidity Index (CCI) to collect comorbidity details up to 6 months prior to the index date [16]. To remove potential symptoms of MM, a 6-month washout period was set. Additionally, for each comorbidity, the primary diagnosis or at least two entries for sub-diagnoses before the index date were required, with the initial diagnosis date being that of the comorbidity. Each comorbidity was defined using ICD-10 codes. For example, congestive heart failure (I09.9, I11.0, I13.0, I13.2, I25.5, I42.0, I42.5–I42.9, I43.x, I50.x, or P29.0); peripheral vascular disease (I70.x, I71.x, I73.1, I73.8, I73.9, I77.1, I79.0, I79.2, K55.1, K55.8, K55.9, Z95.8, or Z95.9); dementia (F00.x–F03.x, F05.1, G30.x, or G31.1); chronic pulmonary disease (I27.8, I27.9, J40.x–J47.x, J60.x–J67.x, J68.4, J70.1, or J70.3); autoimmune disease (M05.x, M06.x, M31.5, M32.x–M34.x, M35.1, M35.3, or M36.0); peptic ulcer disease (K25.x–K28.x); diabetes with (E10.2–E10.5, E10.7, E11.2–E11.5, E11.7, E12.2–E12.5, E12.7, E13.2–E13.5, E13.7, E14.2–E14.5, or E14.7) or without chronic complications (E10.0, E10.1, E10.6, E10.8, E10.9, E11.0, E11.1, E11.6, E11.8, E11.9, E12.0, E12.1, E12.6, E12.8, E12.9, E13.0, E13.1, E13.6, E13.8, E13.9, E14.0, E14.1, E14.6, E14.8, or E14.9); hemiplegia or paraplegia (G04.1, G11.4, G80.1, G80.2, G81.x, G82.x, G83.0–G83.4, or G83.9); renal disease (I12.0, I13.1, N03.2–N03.7, N05.2–N05.7, N18.x, N19.x, N25.0, Z49.0–Z49.2, Z94.0, or Z99.2); any malignancy excluding skin cancer (C00.x–C26.x, C30.x–C34.x, C37.x–C41.x, C43.x, C45.x–C58.x, C60.x–C76.x, C81.x–C85.x, C88.x, or C91.x–C97.x); moderate or severe hepatic disease (I85.0, I85.9, I86.4, I98.2, K70.4, K71.1, K72.1, K72.9, K76.5, K76.6, or K76.7); metastatic solid tumor (C77.x–C80.x); and AIDS/HIV (B20.x–B22.x, or B24.x). In defining ‘any cancer’ for this study, we included any malignancy excluding skin cancer and considered metastatic solid tumors as separate occurrences. The study also considered socioeconomic status, represented by a numeric value derived from the average monthly insurance premium in the KNHIS database. This status was initially categorized into 11 groups, which included a medical beneficiary set and ten dual score level groups. For analytical purposes, these were then grouped into two major categories: the lower 1st to 3rd percentiles (scores 0–2) and the 4th to 10th percentiles (scores 3–20).

### Outcomes

The primary outcome measure was the incidence of CV events among individuals who survived long-term following the index date. We determined the incidence of CV events using specific ICD-10 codes. These included codes for myocardial infarction (I21.x, I22.x, I24.x, or I25.2) and cerebrovascular disease (G45.x, G46.x, H34.0, or I60.x–I69.x). A CV event was recognized if it was recorded as either the primary or secondary diagnosis in the medical expense claims submitted to the KNHIS until the end of the follow-up period, which was on December 31, 2020.

### Propensity score matching

To account for baseline characteristic differences between the case and control group participants, we employed PSM. The PS was derived using a logistic regression model, which factored in variables such as age, sex, index year, socioeconomic status, and a range of comorbidities. These comorbidities included congestive heart failure, peripheral vascular disease, chronic pulmonary disease, autoimmune disease, peptic ulcer disease, diabetes, hemiplegia or paraplegia, renal disease, hepatic disease, and any type of cancer. We only included CCI variables with a prevalence of 0.1% or higher as conditioning variables in the model. For constructing the final study population, we used 1:1 PSM without replacement, employing a greedy-matching algorithm with a caliper width of 0.25. To evaluate the effectiveness of the matching process, we calculated the standardized mean differences (SMDs) of each covariate across the groups both before and after applying PSM. The matching was considered balanced and effective when the SMD for each covariate was less than 0.1 [17].

### Statistical analyses

Continuous variables are expressed as means ± standard deviations, and categorical variables are presented as frequencies or percentages. We measured the time-to-event from the baseline until a CV event diagnosis or death, whichever came first. Patients who did not experience a CV event or die during follow-up were censored at their last medical encounter. The cumulative incidence of CV events was calculated, accounting for death as a competing event, and the difference in cumulative incidence between the MM and non-MM groups was tested using the Gray test static for equality. Instead of the traditional Cox proportional hazards model, which typically treats death as censored, we employed the Fine–Gray model. This model considers death as a competing risk, providing a more conservative estimate than methods that treat patients who die as censored, assuming they would remain at risk with more extended follow-up. To account for the clustering of matched pairs, statistical inference was based on the Fine–Gray proportional hazard model with robust standard errors using the sandwich covariance matrix estimation. The hazard ratio (HR) and its 95% confidence interval (CI) were estimated using Fine–Gray models. We implemented a five-year landmark analysis to address immortal time bias in survival analysis, focusing on long-term survivors to identify CVD events. All tests were two-tailed, and statistical significance was set at *P* < 0.05. Analyses were conducted using SAS version 9.4 (SAS Institute Inc.) and R version 4.0.3 (R Foundation for Statistical Computing, Vienna, Austria).

## Results

### Baseline characteristics

**Fig. 1:**
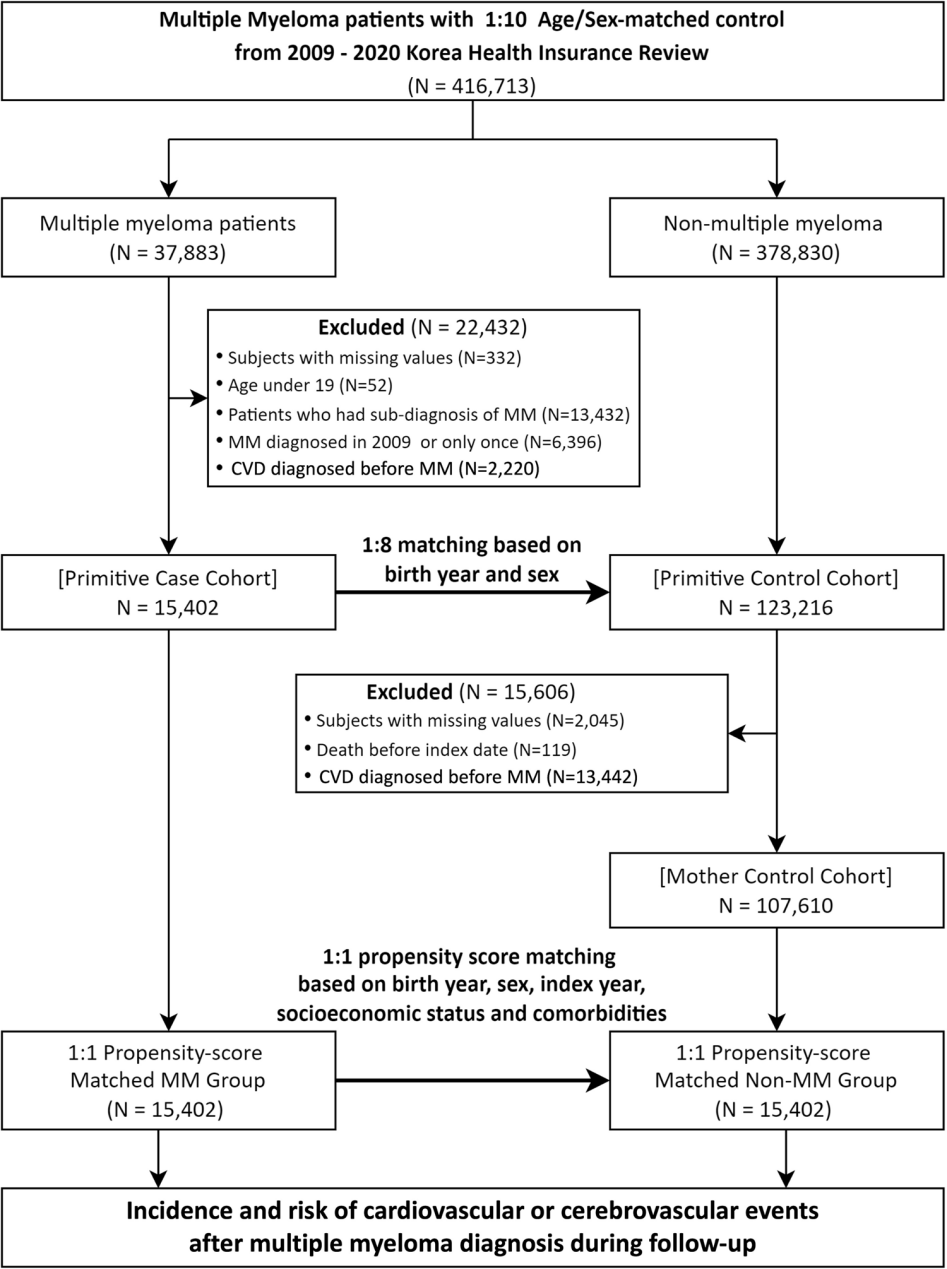
Flowchart illustrating the study population selection process

Table 1 represents the baseline characteristics of the study cohort before and after PSM. Before PSM, we identified a total of 15,402 patients with MM (case cohort) and 107,610 healthy controls (control cohort) who had not been diagnosed with MM. Before PSM, the case cohort was older and showed differences in comorbidities compared with the control cohort. After applying 1:1 PSM, the two groups were balanced, with each group containing 15,402 individuals. The SMDs for all matched variables were effectively balanced, with most showing an SMD of < 0.1. The mean age in the case cohort was 66.2 ± 11.5 years, compared with 66.1 ± 11.3 years in the control group. No differences were observed in follow-up duration, gender ratio, socioeconomic status, or comorbidities between the two cohorts (Fig. 1).

**Table 1:** Baseline characteristics of study population before and after propensity score matching

	Before propensity score matching			After propensity score matching^a^		
	Case cohort (n = 15,402)	Control cohort (n = 107,610)	SMD	Case cohort (n = 15,402)	Control cohort (n = 15,402)	SMD
Follow-up duration^b^ (years)	2.2 [0.8–4.4]	5.0 [2.4–7.8]		2.2 [0.8–4.4]	4.9 [2.3–7.7]	
Age (years)			0.08			0.03
< 60	4246 (27.6%)	32,418 (30.1%)		4246 (27.6%)	4189 (27.2%)	
60–69	4675 (30.4%)	33,521 (31.2%)		4675 (30.4%)	4802 (31.2%)	
70–79	4683 (30.4%)	30,676 (28.5%)		4683 (30.4%)	4711 (30.6%)	
≥ 80	1798 (11.7%)	10,995 (10.2%)		1798 (11.7%)	1700 (11.0%)	
Mean ± SD	66.2 ± 11.5	65.3 ± 11.5	0.08	66.2 ± 11.5	66.1 ± 11.3	< 0.01
Sex			< 0.01			< 0.01
Female	7058 (45.8%)	49,435 (45.9%)		7058 (45.8%)	7048 (45.8%)	
Male	8344 (54.2%)	58,175 (54.1%)		8344 (54.2%)	8354 (54.2%)	
Socioeconomic status			0.05			< 0.01
Low	2769 (18.0%)	17,183 (16.0%)		2769 (18.0%)	2761 (17.9%)	
Middle-high	12,633 (82.0%)	90,427 (84.0%)		12,633 (82.0%)	12,641 (82.1%)	
Comorbidity						
CHF	1199 (7.8%)	5,864 ( 5.4%)	0.09	1199 (7.8%)	1224 (7.9%)	< 0.01
PVD	3414 (22.2%)	20,489 (19.0%)	0.08	3414 (22.2%)	3375 (21.9%)	< 0.01
Dementia	654 (4.2%)	4103 (3.8%)	0.02	654 (4.2%)	654 (4.2%)	< 0.01
Hemiplegia or paraplegia	73 (0.5%)	496 (0.5%)	< 0.01	73 (0.5%)	81 (0.5%)	< 0.01
Autoimmune disease	1266 (8.2%)	6972 (6.5%)	0.07	1266 (8.2%)	1290 (8.4%)	< 0.01
CPD	7166 (46.5%)	42,299 (39.3%)	0.15	7166 (46.5%)	7129 (46.3%)	< 0.01
Peptic ulcer disease	6795 (44.1%)	41,769 (38.8%)	0.11	6795 (44.1%)	6702 (43.5%)	0.01
Hepatic disease	4520 (29.3%)	27,823 (25.9%)	0.08	4520 (29.3%)	4563 (29.6%)	< 0.01
Renal disease	569 (3.7%)	1,617 (1.5%)	0.14	569 (3.7%)	560 (3.6%)	< 0.01
Diabetes	3951 (25.7%)	23,612 (21.9%)	0.09	3951 (25.7%)	3940 (25.6%)	< 0.01
Any cancer	1574 (10.2%)	8,417 (7.8%)	0.08	1574 (10.2%)	1544 (10.0%)	< 0.01
AIDS/HIV	3 (0.0%)	20 (0.0%)	< 0.01	3 (0.0%)	2 (0.0%)	< 0.01

SMD, standardized mean difference; MI, myocardial infarction; CHF, congestive heart failure; PVD, peripheral vascular disease; CVD, cerebrovascular disease; CPD, chronic pulmonary disease; a, the propensity score model included age, sex, index year, socioeconomic status, and prior diseases conditions; b, follow-up time represented as median [IQR]; continuous variables are presented as mean ± standard deviation(SD); categorical variables are presented as n (%); control cohort, non-multiple myeloma group; case cohort, multiple myeloma group

### Incidence of cardiovascular or cerebrovascular disease after multiple myeloma diagnosis

**Fig. 2:**
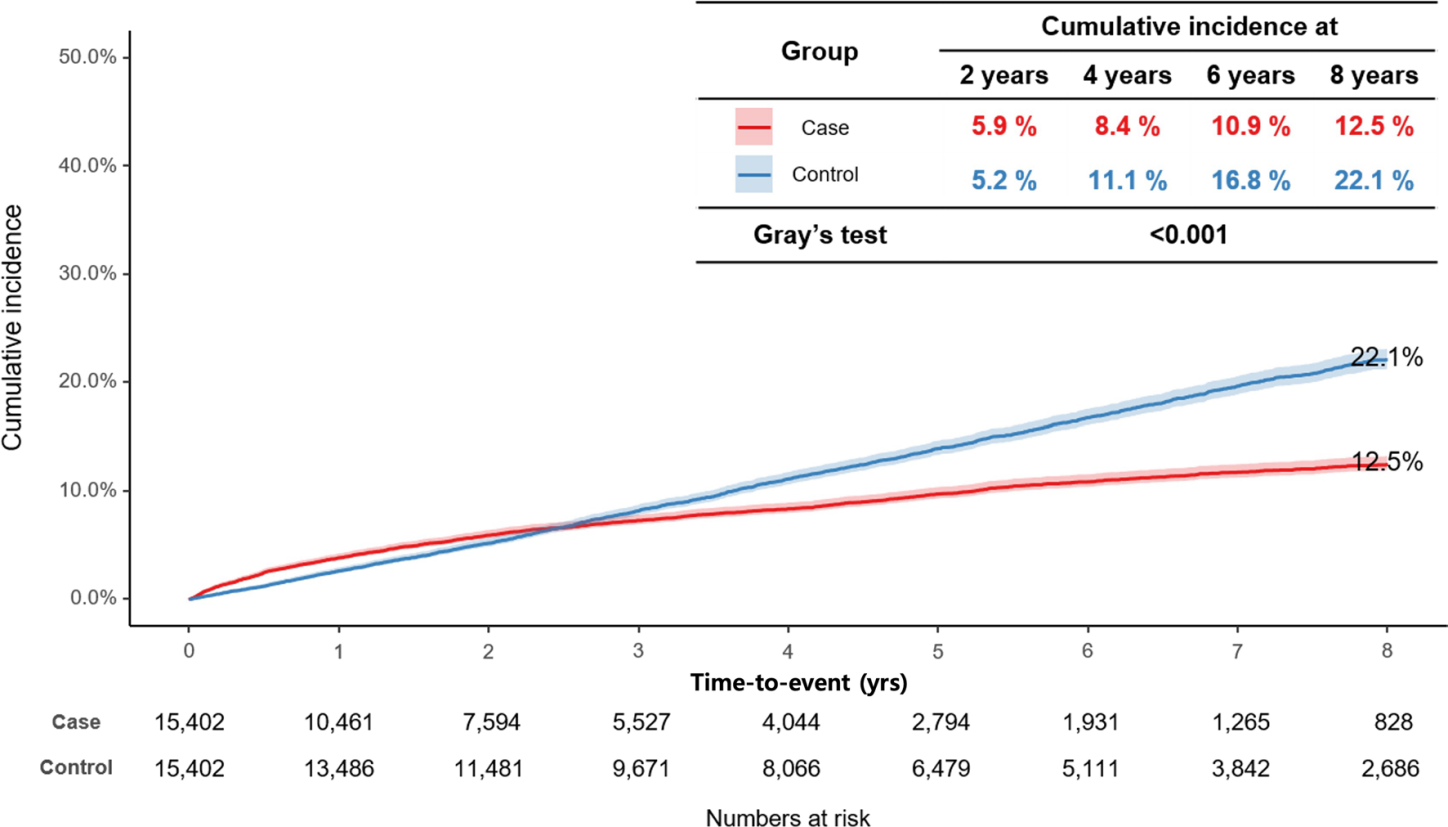
Cumulative incidence of cardiovascular disease (CVD) events in the case and control cohorts during follow-up. Shaded areas represent the 95% confidence interval (CI)

The risk of CVD, as evidenced by the cumulative incidence, differed significantly between the case and control cohorts (Gray’s test *P* = 0.001). By the 8-year follow-up, the cumulative incidence of CVD in the case cohort was 12.5%, compared with 22.1% in the control cohort (Fig. 2). The incidence of CVD was elevated in the case cohort for approximately the first 2.5 years, with a 2-year cumulative incidence of 5.9% compared with 5.25% in the control cohort. However, beyond this period, the incidence in the case cohort consistently remained lower than that in the control cohort.

### Incidence of CVD after multiple myeloma diagnosis: 5-year landmark analysis

**Fig. 3:**
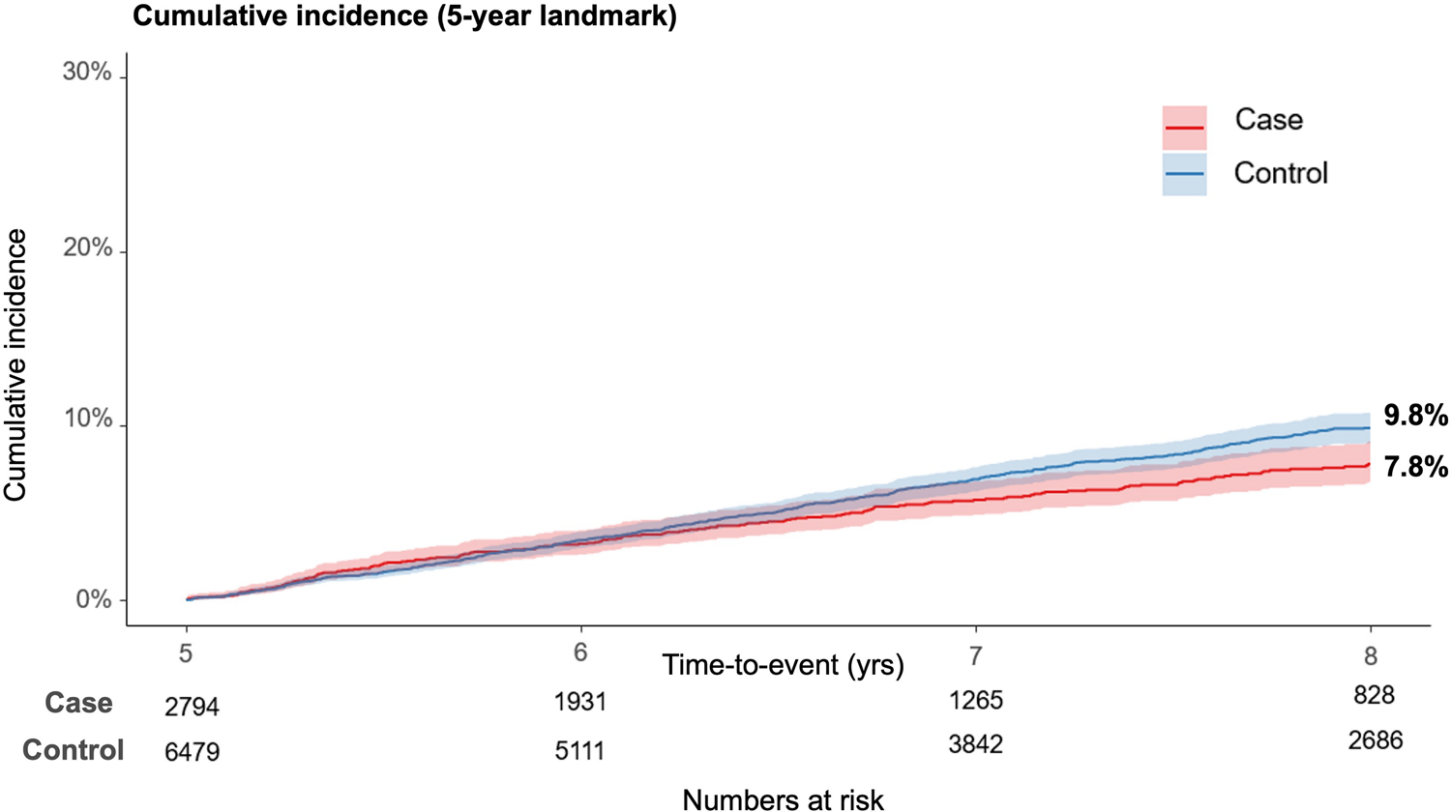
Five-year landmark analysis of cardiovascular disease (CVD) events in the case and control cohorts. Shaded areas represent the 95% confidence interval (CI)

**Fig. 4:**
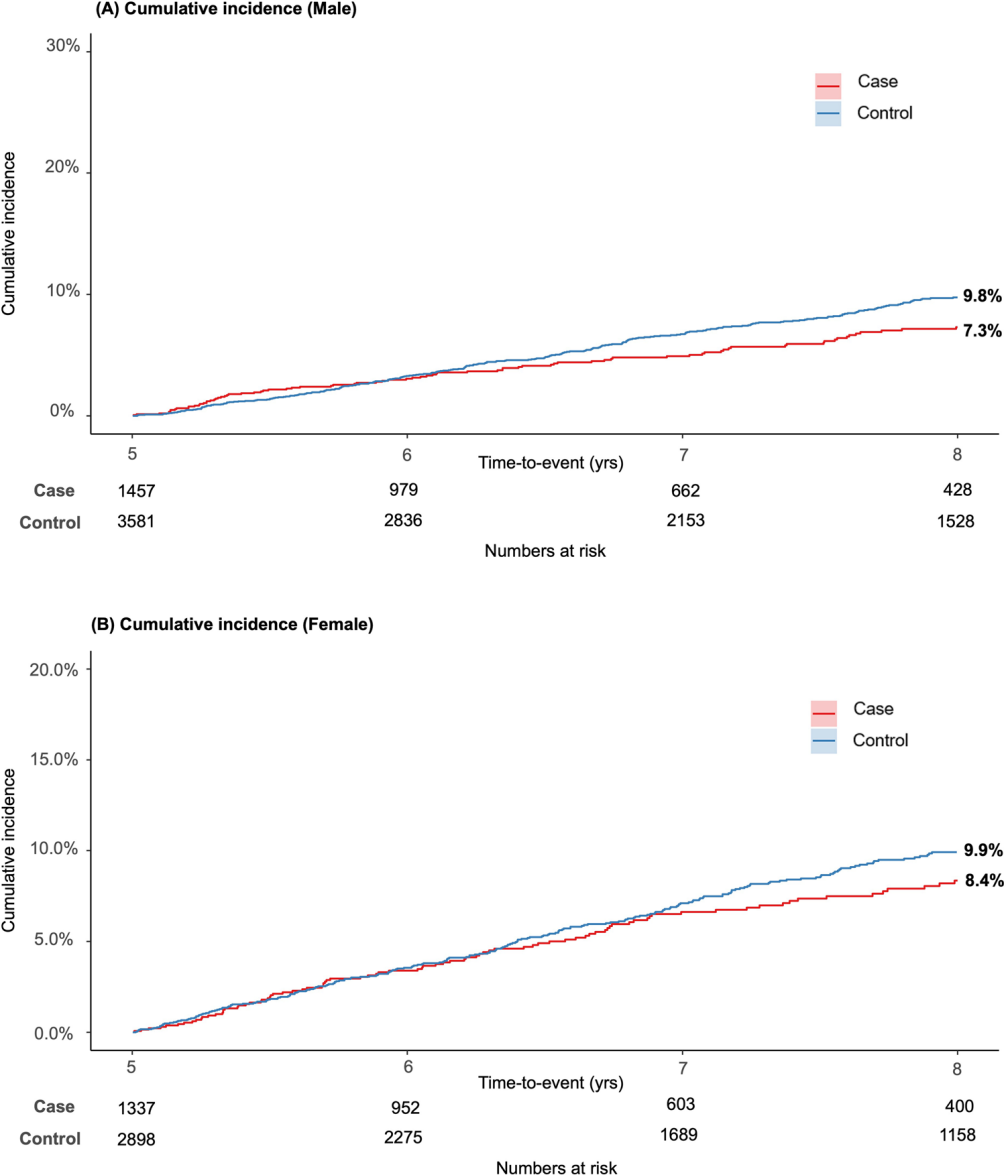
Five-year landmark analysis of cardiovascular disease (CVD) events in the case cohort compared with the control cohort, stratified by sex

**Fig. 5:**
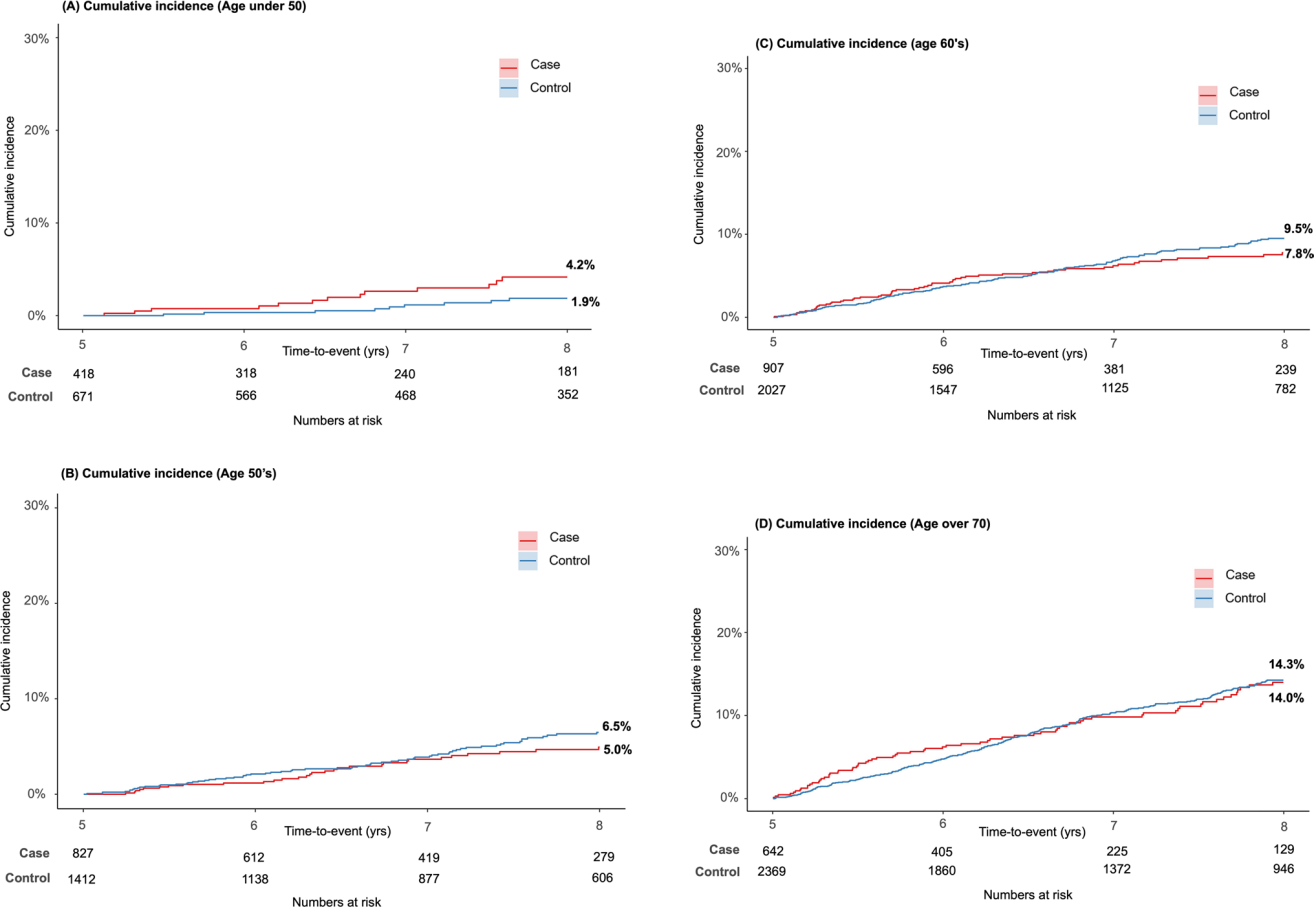
Five-year landmark analysis of cardiovascular disease (CVD) events in the case cohort compared with the control cohort, stratified by age

The results from the 5-year landmark analysis of CVD events show a significant difference in cumulative incidences between the cohorts (Fig. 3). After applying the 5-year landmark analysis, cumulative incidences differed: 7.8% and 9.8% for the case and control cohorts, respectively (Fig. 3). The statistical significance of this difference was confirmed using Gray’s test, with a *P*-value of < 0.001. This pattern of reduced CVD risk observed in the case cohort was consistently replicated in the comparative analysis between men and women (Fig. 4). Age group analysis revealed variations in the occurrence of CVD events between the cohorts (Fig. 5). The incidence of CVD was significantly lower in the case cohort among individuals in their 50 s and 60 s (Figs. 5B and C), whereas no significant difference was observed in those aged 70 years and above (Fig. 5D). Notably, the incidence of CVD among individuals aged under 50 years in the case cohort (4.2%) was significantly higher than that in the control cohort (1.9%, *P* < 0.001) (Fig. 5A).

## Discussion

This cohort study conducted a retrospective analysis of CVD events in patients with MM, mainly focusing on the prevalence of CVD among long-term survivors. The findings revealed a lower incidence of CVD events among these long-term survivors than that in the non-MM group. These patterns were consistent across analyses of both sex and age groups 50 years and older. However, the incidence of CVD was consistently higher among patients with MM aged < 50 years at diagnosis.

Existing reports indicate that patients with MM generally experience an increased incidence of CVD events [13, 18]. Age is a well-known risk factor for CVD, and given that MM predominantly occurs in older adults [9], treatments for MM—such as cardiotoxic chemotherapy, high doses of steroids, proteasome inhibitors, immunomodulatory drugs, and radiotherapy—may further escalate the risk of CVD [7, 19]. The underlying mechanisms, still not fully understood, may involve (1) myocardial cell damage from reactive oxygen species [20]; (2) significant structural changes in cardiomyocyte mitochondria, reducing cardiac contractility [21]; and (3) a decrease in tissue nitric oxide levels, resulting in endothelial dysfunction [21]. In addition, a range of patient- and disease-related factors, including male sex and renal dysfunction, have been identified as risk factors for CVD events in patients with MM. These findings indicate that these events result from multiple contributing factors [18, 22, 23].

In phase 3 trials, where the median overall survival was under 4 years, approximately 7.5% of patients with MM experienced CVD [14]. The present study reported a comparable 4-year CVD incidence rate of 8.4%. However, this study focused on patients with relapsed or refractory MM, potentially not capturing the full scope of long-term CVD incidence among survivors.

The investigation unveiled a nuanced pattern in the incidence of CVD events among patients diagnosed with MM. Initially, within approximately 2.5 years following diagnosis, the cohort of patients with MM exhibited a significant increase in the risk of CVD events compared with the control group. This initial heightened risk may be attributed to an array of well-documented factors. This also aligns with earlier research indicating that non-cancer-related issues, such as CV mortality, are common in the first year following a cancer diagnosis, potentially as a result of treatment [24–28]. Subsequently, an observable decline in the risk of CVD events was noted, particularly pronounced among long-term survivors of MM, defined as individuals surpassing the five-year survival threshold. This longitudinal shift suggests a complex interplay of pathophysiological, therapeutic, and possibly adaptive mechanisms influencing CV risk profiles over time in this patient population. Rosenberg et al. reported the incidence of major CV events in patients with MM over time [29]. Contrary to expectations, they found that the incidence of CVD events in patients with MM has actually decreased in recent years compared with that in the 1990s. In population-based studies, mortality, including age-adjusted CV mortality, in patients with MM has continued to decline in recent years [10]. Identifying the causes behind these findings is challenging. Nonetheless, the observed decrease in the risk of CVD events among patients with MM over time might be attributed to heightened vigilance regarding CV health in this demographic or the preemptive employment of interventions, including the use of anti-platelet agents [29]. Rosenberg et al. highlighted that heightened awareness of CVD in patients with MM has prompted more proactive screening and intervention efforts, potentially lowering the risk of CVD in long-term survivors [29]. This approach, involving periodic screening along with timely and suitable interventions, could have played a significant role in diminishing the risk of CVD events.

A steady decrease in CVD events over time after MM diagnosis was observed. This trend persisted across analyses by sex and was observed in most age groups in this study. Notably, patients diagnosed with MM at a younger age, particularly those aged under 50 years, had a higher risk of experiencing CVD. In patients diagnosed with MM before the age of 50 years, the cumulative 8-year CVD event rate was 4.2%, compared with 1.9% in the control group. This study employed a 5-year landmark analysis to minimize immortal time bias. While this bias is anticipated to have a lesser impact on younger patients under the age of 50 years, the clinical relevance of an increased cumulative incidence of CVD over time remains substantial. Similar to our results, Yin et al. studied 88,328 patients with MM and found that those aged under 50 years at diagnosis had the highest CVD mortality rates among the age groups compared [10]. This is consistent with previous findings that younger cancer patients have higher all-cause mortality rates [24, 30–32]. We theorize that the observed patterns may be attributed to several factors: (1) the lower prevalence of CVD in younger individuals might lead to an overestimation of its occurrence in the younger MM population; (2) the incidence of CVD might be underestimated in older patients, who have a higher likelihood of dying from other causes; and (3) younger individuals are less inclined to use CVD-reducing medications, such as statins, compared to their older counterparts. This study indicates that a diagnosis of MM at a younger age may correlate with a heightened risk of CVD events, even among those who achieve long-term survival. Therefore, proactive evaluation and monitoring for CVD events are advisable, along with the diligent use of agents known to mitigate the risk of such events, including statins.

The present study has certain limitations. First, the estimation of CVD events was based on hospital records, not confirmed diagnoses. Despite the Korean public insurance system’s comprehensive use of ICD-10 codes from medical records, which are stringently checked, discrepancies can occur. Second, our dataset did not include clinical specifics regarding patients with MM, such as remission status, medication types, nutritional health, social habits, and other pertinent MM-related characteristics. This constraint hampers the analysis of CVD occurrence in relation to each patient’s comorbidities and specific CVD risk factors. While our analysis could not encompass every factor contributing to the development of CVD, it is important to highlight that this study, grounded in real-world conditions, discerned patterns of CVD occurrence among long-term survivors of MM. Third, our analysis could have been influenced by immortal time bias. To counteract this, we implemented time-dependent covariates and landmark analysis, thereby enhancing the reliability of our findings. Establishing a clear link between CVD events and mortality proved challenging. Further long-term studies are necessary to ascertain whether the elevated risk of CVD events leads to higher mortality rates, particularly among younger patients aged under 50 years.

To the best of our knowledge, this is the first comprehensive national case–control study in Korea to investigate the incidence of CVD in patients with MM, particularly those with long-term survival exceeding 5 years. The observed trend indicated a reduction in the risk of CVD events among long-term survivors of MM, except for individuals aged under 50 years at diagnosis, who experienced an elevated risk. These findings suggest that clinicians should consider active monitoring for CVD events as an essential part of long-term care, particularly for patients aged under 50 years at first MM diagnosis.

## Data Availability

Some or all data sets generated during and/or analyzed during the current study are not publicly available but are available from the corresponding author on reasonable request.
